# The use of green synthesized TiO_2_/MnO_2_ nanoparticles in solar power membranes for pulp and paper industry wastewater treatment

**DOI:** 10.1038/s41598-024-85075-8

**Published:** 2025-01-15

**Authors:** Sahar A. Mousa, Heba Abdallah, S. A. Khairy

**Affiliations:** 1 Physics Department, Faculty of Science, Cairo University, Giza, 12613 Egypt; 2 Chemical Engineering Department, Engineering and Renewable Energy Research Institute, National Research Centre, 33 El-Bohouth St. (Former El-Tahrir St.), Dokki, PO Box 12622, Giza, Egypt

**Keywords:** Photocatalytic membrane, PVC membrane, Heterostructure, TiO_2_, Green synthesized, Pulp and paper wastewater, And TiO_2_/MnO_2_ nanocomposite, Materials science, Nanoscience and technology, Physics

## Abstract

The pulp and paper manufacturing wastewater is as complicated as any other industrial effluent. A promising approach to treating water is to combine photocatalysis and membrane processes. This paper demonstrates a novel photocatalytic membrane technique for solar-powered water filtration. The method is based on creating green-prepared TiO_2_, and MnO_2_ nanoparticles (NPs) using Pomegranate peels and Seder leaf extracts and incorporation into polyvinylidene chloride to produce a novel water purification system that combines semiconductor photocatalysis with membrane filtration. The prepared heterostructure of the TiO_2_/MnO_2_ nanocomposite membrane provides photogenerated charge separation. To ensure chemical bonding at the membrane surface, Raman and Fourier transform infrared spectroscopy (FT-IR) were employed. The modified membrane’s hydrophilicity and roughness increased significantly. Additionally, the modified nanocomposite membrane^’^s porosity was measured. The integrated process demonstrated much higher removal of humic acid and high efficiency of wastewater treatment for pulp and paper. In sunlight, humic acid removal was 98% from synthetic wastewater. While using the produced membrane on pulp and paper effluent, these studies indicate that: in the dark, the removal was 50%, while in the sunlight, the removal increased to 70%, with a reduction in the COD from 1500 mg/L to 247 mg/L. Additionally, the TDS decreased from 1630 to 452 ppt in the sunlight. This research sheds light on how solar energy can clean wastewater from the pulp and paper industry while improving membrane separation. Also, an alternative source to sunlight was used to manufacture a photocatalytic membrane with high efficiency for wastewater treatment and an inexpensive price.

## Introduction

Water is one of the most fundamental necessities of existence. It is unimaginable for people to live without water. Nowadays, the world’s cities are increasing faster than ever before, and there is not enough water available in sufficient quality or quantity^1^. More liquid wastes from anthropogenic and other environmental activities end up in water supplies where they are dumped. The main sources of contaminants in wastewater are home and industrial wastes composed of agricultural practices. Polyphenols, halogenated compounds, organic acids, fertilizers, dyes, heavy metals, pesticides, and microbes are among the hazardous elements found in wastewater. Using polluted water directly poses several major health hazards^2,3^. The sources of a few prevalent water contaminants are listed^4,5^. The globe is running out of fresh water, and it’s not looking good for the future. To ensure that clean water is available to all living creatures, it is crucial to eliminate these toxins. By removing all pollutants and toxins, wastewater treatment improves and purifies water, ready for reuse or release into the environment^6,7^.

The paper and pulp industry is one of the largest consumers of water and mineral resources, and as a result, it generates a lot of contaminated water from numerous unit operations^8,9^. After the steel, cement, leather, textile, and oil sectors, it is the sixth largest polluter of the environment, releasing a variety of wastes into the atmosphere.^10^. The paper and pulp industry uses a significant amount of energy in its manufacturing process since energy costs account for more than 13% of all production costs.^11^. This industry uses a lot of freshwater (60–230 m^3^ for every ton of paper produced), which results in the production of a large amount of effluent (at least 50 m^3^)^12–15^. According to earlier research, wastewater from the pulp and paper sector continues to be a source of pollution even after secondary treatment. The receiving water’s physicochemical parameters, such as total suspended solids (TSS), chemical oxygen demand (COD), total dissolved solids (TDS), biological oxygen demand (BOD), metals (e.g., Fe, Cu, Ni, Cr, Pb, Cd, Hg, As, Mg, and Mn), and other organic pollutants, are increased, which poses a direct and indirect hazard to microorganisms and aquatic organisms. There are now many different techniques used to treat industrial effluents from the pulp and paper industry, comprising aerobic, photocatalysis, electrochemical, anaerobic, coagulation-flocculation, adsorption behavior, and ozonation, procedures^16–21^.

Water treatment methods that are safe, economical, and efficient are still in great demand but difficult to implement^22,23^. Membrane filtration has garnered significant interest due to its rapid pollution separation and minimal environmental impact^24,25^. Microfiltration has been recognized as a promising technique due to its low energy consumption and excellent manufacturing yield^26,27^. However, in the basic separation process, many organic pollutants with smaller sizes than their pores are not removed by commonly employed microfiltration membranes based on size exclusion, which can lead to potentially hazardous effluent water. Furthermore, its application is limited by the intrinsic membrane fouling, which raises permeate loss and energy costs^28–30^.

Because of its remarkable mechanical strength, resistance to acid, alkali, microbiological agents, and affordability, poly (vinyl chloride), sometimes known as PVC, is a highly recommended membrane material. Unfortunately, because of their intrinsic low flux and fouling susceptibility, PVC membranes have a limited range of uses. These membranes’ performance and antifouling characteristics are currently being worked on extensively. When making PVC-based membranes, one of the most communal approaches for improving or refining is blending and adding hydrophilic additives to the incapacitated solution before casting. An uncomplicated but dependable method is blending^31–38^. Because feed fluids are complicated and diverse membrane as a result of the complication and diversity of feed fluids, membrane fouling regulation is motionless and constrained in standings of outward functionality, such as operating conditions. Self-cleaning and efficient membranes that can break down foulants on the membrane surface or catalyze processes that degrade foulants have drawn more attention. To achieve the simultaneous filtering and annihilation of organic pollutants, photocatalytic activity is combined with the membrane material to generate a photocatalytic membrane (PCM)^39^.

The synthetic processes used to produce nanoparticles (NPs) are expensive and harm the environment. As a result, using greener, plant-assisted nanomaterials is thought to be preferable to chemical procedures. To reduce the amount of harmful reagents used during the synthesis process, biosynthesis is a very useful method for producing nanoparticles (NPs) since it uses inexpensive, sustainable, and biologically produced precursors^40^. Three main categories of biological sources, including plant extract, enzymes, and bacteria, are usually used in the biosynthesis process^41^. Plant extracts are abundant and reasonably priced, making their use in the production of nanoparticles a highly feasible method. Furthermore, due to their environmentally friendly nature, plant-based NP synthesis methods are widely used and are easy to scale up. Effective reducing properties are exhibited by plant metabolites, including phenolic compounds and alkaloids. Varied Nanoparticles were prepared using different plant extracts. Pomegranate extract was used to prepare a lot of NPs such as ( Ag^42,43^, Fe_2_O_3_^44,45^, ZnO^46,47^, and TiO_2_^48^). Seder plant was used also in the preaparation of SiO_2_^49^, Au^50^, and NiO^51^.

The primary drawbacks of membrane processes are their short lifespan and the requirement for a pretreatment system to prevent membrane fouling. Furthermore, operational failures might affect the entire system quite sensitively. Nonetheless, major advancements in membrane technology have been made to provide an overall higher performance. In earlier research, Liu et al. 2020^52^ Prepare the PVDF-Ni-ZnO composite membranes with photocatalytic and magnetic properties for antifouling. The composite PVDF-NZPs membrane demonstrated enhanced antifouling capabilities, magnetic characteristics, and photocatalytic qualities as compared to the bare PVDF membrane. Furthermore, the modified PVDF-NZPs membrane showed improved antifouling capabilities with a comparatively high flux and high FRR (100%, 100%, 83%, and 83% for the filtration of HA, SA, BSA, and yeast, respectively). Rathna et- al 2021^53^ Prepare TiO_2_-WO_3_(40:1) nanoparticles, implant them in Polyaniline (PANI) membrane and apply them in photocatalytic membrane reactors to remove Cr(VI) from aqueous solutions at different concentrations (3–7 wt%). Under visible light, the 5 wt% TiO_2_-WO_3_- PANI membrane displayed a 67.32% decrease. Dalanta et al. 2022^54^ attention is given to the effects of a cobalt-doped TiO_2_@SiO_2_ on polysulfone (PSf) photocatalytic membranes for treating petroleum refinery wastewater under UV light. Regarding durability, stability, recycling capacity, permeate flux, and pollutant rejection, the PSf/Co-TiO_2_@SiO_2_ membrane performed better than expected. Co-doping in TiO_2_ effectively increased the composite’s photosensitivity and photocatalytic activity. Kusworo et- al 2023^55^ constructed a MnO_2_@SiO_2_ membrane to treat rubber-laden wastewater treatment by utilizing the non-solvent induced phase separation method to add a photocatalyst composite of manganese oxide and ZnO at SiO_2_ (ZnO-MnO_2_@SiO_2_) to a polysulfone (PSf) membrane. The UV light of the PSf/ZnO-MnO_2_@SiO_2_ membrane considerably increased the flux stability, membrane reuse, and antifouling capabilities, which reduced fouling formation and extended the membrane’s usefulness. This is understandable given the modified membrane’s high photocatalytic activities and the interaction energies connected to different foulants’ adherence. The research and its novelty aim to develop photocatalytic membrane systems made of polyvinyl chloride and nanoparticles manufactured from plant leaves. This study clarifies how solar energy might enhance membrane separation and clean effluent from the pulp and paper industry. A streamlined system setup, financial gains, and environmental sustainability are just a few of the benefits that come with this integration. An alternative source for the sun was selected to facilitate the creation of a photocatalytic membrane that was cheap and did not require large areas**.**

## Experimental part

### Materials and methods

#### Materials

Qualikem Fine Chem Pvt. Ltd. (India) supplied N-dimethylacetamide (DMAc, 99%), polyvinylpyrrolidone (K25) N, and high molecular weight (40000 g/mol) polyvinyl chloride (PVC), and Loba Chemie (India) supplied titanium tetrachloride (TiCl_4_), humic acid (60%) (Loba), and permanganate potassium KMnO_4_ (98% purity), Seder leaves, and pomegranate fruits.

### Nanoparticles production

#### Seder leaves extract

A precise cleaning process using flowing water was used to eliminate dust from 140 g of freshly harvested Seder leaves. A series of DW rinses were then applied to the leaves. A pale-yellow solution was obtained by boiling the leaves for 120 min with 700 mL of DW. It was allowed to reach room temperature. The solid ingredients in the extract were then filtered out using Wattman filter sheets^56^.

#### Pomegranate extract

Dust was removed from the pomegranate peels by repeatedly washing them under running water. Then, the pomegranate was repeatedly cleaned using DW. Cut into small pieces, 240 g of fresh pomegranate peels were heated in 1.2 L DW till boiling, then allowed to cool at ambient temperature. The required extract was obtained by filtering the extract using Wattman filter sheets once it had cooled^56^.

#### Preparation of TiO_2_ NPs

A 0.5 L amount of pomegranate peel extract was agitated for half an hour and then refrigerated. To the beaker holding the plant extract, a suitable amount of TiCl_4_ was gradually added. For an entire hour, the reaction was immersed in an ice bath. Following that, After being raised to 80 °C, the temperature remained there for two hours. DW was used to wash the TiO_2_ NPs many times before they were dried at 70 °C. After that, the powders were calcined at 450 °C for three hours^57^.

#### Preparation MnO_2_ NPs

After 30 min of stirring 500 ml of Seder extract, 20 g of KMnO_4_ was progressively added. The 30-min reaction was maintained under the same circumstances. The temperature was then increased to 80ºC. For two hours, the solution was maintained in these circumstances. The particles were dried at 80 °C after being washed several times with DW. Following that, it was burned and calcined for 180 min at 450 °C^58^.

### Synthesis of TiO_2_/MnO_2_ photocatalytic membranes

**Fig. 1:**
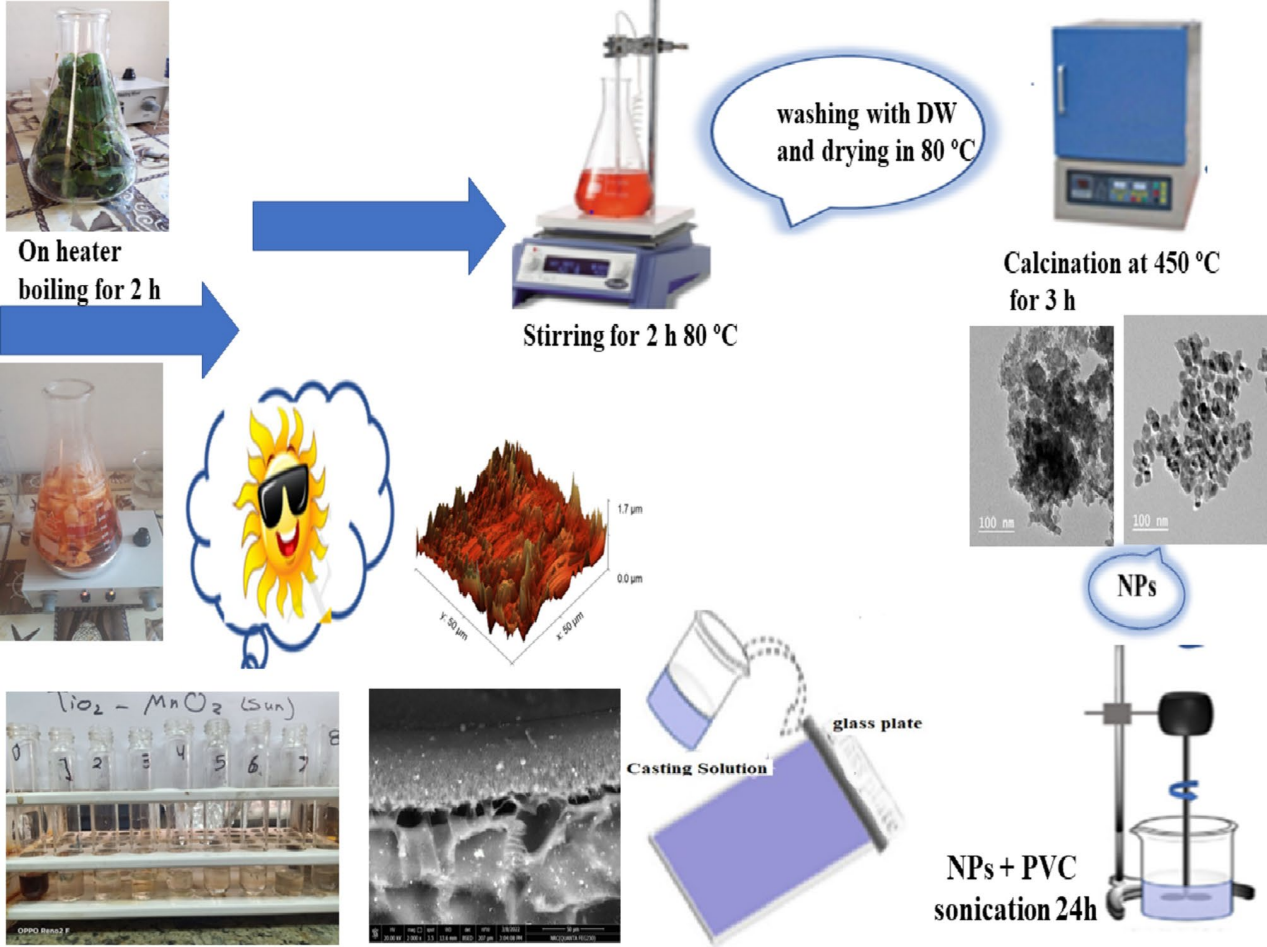
Preparation process of the TiO_2_/MnO_2_ photocatalytic membrane.

Phase inversion process was handled to construct photocatalytic TiO_2_ / MnO_2_ photocatalytic membranes. Figure 1 illustrates the stages of preparation. Initially, 2 g of NPs (1 g TiO_2_ + 1 g MnO_2_) were ultrasonically distributed in 84 g of DMAc for one hour. To create a uniform casting solution, 12 g of PVC was dissolved in the mixture while 2 g of PVP was supplementary and vigorously stirred. Once the slurry has been degassed, A 200 µm-thick casting knife is used to deposit it onto a glass plate, that is gently run over a nonwoven fiber. Without the solvent evaporating, the cast sheets were submerged in a coagulation bath (DW) to finish the solvent/nonsolvent separation. The produced membrane was allowed to fully coagulate, rinsed in DW several times to eliminate any remaining solvent, and then saved in DW until further application.

### Characterization

#### Nanoparticles characterization

The structure and crystallinity of the generated NPs were identified using a D8-Advance Bruker AXS diffractometer. The Scherrer equation, Eq. (1), was used to get the average crystal size.

1 $$\text{D }= \frac{0.9.\uplambda }{\upbeta .{{\rm cos}\theta }}$$

The X-ray wavelength is represented by λ, ϴ is the Bragg diffraction angle (°), When using complete width at half maximum, the Scherer factor (0.9) and the half-height width (rad) of the strongest diffraction peak have been included in the calculation. Equation (2) was used to estimate the mass fraction of the anatase phase to the rutile phase W_R_. where A_A_ and A_R_ stand for anatase (101) and rutile (110) intensities, respectively^56^

2 $${\text{W}}_{{\text{R}}} = {\text{ A}}_{{\text{R}}} / \, \left( {0.886{\text{A}}_{{\text{A}}} + {\text{ A}}_{{\text{R}}} } \right) \, \%$$

The dislocation density (δ) and the microstrain $(\varepsilon )$ were estimated according to Eqs. (3, 4).

3 $$\varepsilon =\frac{\beta cos\theta}{4}$$

4 $$\delta = \frac{1}{D2}$$

Their morphology was described using high-resolution transmission electron microscopy (HRTEM), which was executed by expending a TEM-1230 (JEOL Co., Japan) at an accelerating voltage of 100 kV. To find the energy bandgap of NPS, the diffuse reflectance UV–Vis spectroscopy was required.

#### Membrane characterization

The FE-SEM**,** or field emission scanning electron microscopy model, JSM-T20 JEOL, Japan, was used to describe the surface morphologies and cross-sectional features of the constructed membrane. The Topography and the degree of roughness were studied using AFM (atomic force microscope; Agilent Technologies, 5600 LS) that was used for the analysis of the topography and the surface roughness of the upper membrane layers. A little piece for every membrane was sliced and placed on the highest point of a circular sample container. that measured one centimeter in diameter.

With the aid of an image-processing software-equipped contact angle goniometer (Attention, Theta, by Biolin Scientific), the contact angle of the membrane was ascertained. This device was utilized to evaluate the hydrophilicity of the inorganic nanoparticles.

To ensure chemical bonding at the membrane surface: PE-100, US, FT-IR (Fourier transform infrared spectroscopy) was used. The specimens were positioned on a sample holder, in addition, All the measured spectra were obtained in the 400–4000 cm^-1^ wavenumber range.

Raman investigations were carried out using a Confocal Raman Microscope (Witec, 300 alpa R, manufactured in Germany). The specimen was excited using a (785 nm) laser light, and readings were taken using the microscope’s beam the path adjusted to 50X.

Porosity and membranes’ water flux: Membrane porosity is calculated by dividing all the volume of the permeable membrane by the size of its pores. The mass of the membrane was evaluated, dried for 24 at 100 °C in an oven, and then soaked in DW for an overnight period at ambient temperature, then dried off and reweighed without somewhat surface moisture that, had been removed using absorbent paper. The membrane porosity ε (%) was ascertained with membrane mass by water uptake experiments and the use of the following equation, Eq. (5)^59^, where the masses of the wet and dry membranes, respectively, are denoted by m_1_ and m_2_. A represents the effective area of the membrane sample under examination, d denotes the membrane’s thickness, and ρ is the density of pure water. To reach the equilibrium swelling state, the prepared membrane was immersed in DW for a whole day before the test.

5 $$\upvarepsilon ({\%})=\frac{\text{m}1-\text{m}2}{\text{A}.\text{d}.\uprho }\times 100$$

Equation (6) was used to calculate the water flux **J** (L\ m^2^.h) of the produced membranes^60^ in which Δt is the time, V is the volume of permeate per unit time (h), and A is correlated with the active membrane area (m).

6 $$\text{J}=\frac{\text{V}}{\text{A }.{ \Delta {\rm t}}}$$

X-ray photoelectron spectroscopy analysis: Utilizing X-ray photoelectron spectroscopy (Fixed Analyzer Transmission, Slit: 4:7 × 20c / C: mesh), the chemical composition of the polymeric membranes’ surfaces was assessed. There was a 280 eV Al Kα radiation source installed in the system. Between 0 and 700 eV, survey spectra were captured. After accounting for atomic sensitivity, the peak area was used to calculate the surface elemental composition:

Surface area measurements: The constructed TiO_2_/MnO_2_ membrane’s surface area was measured. Using a NOVA touch 4LX analyzer (Quantachrome, [s/n:17016062702], USA), Brunauer–Emmett–Teller (BET) measurements were used to determine the surface area and pore size of NP. N_2_ was used in the physisorption investigation at liquid N_2_ temperature. Barrett–Joyner–Halenda (BJH) was used to determine the mean pore size distributions and total pore volume.

### Photocatalytic performance investigation.

The photocatalytic activity of the TiO_2_/MnO_2_ membrane was evaluated in degradation systems under both dark as well as sunlight conditions. For the dynamic system, As the reaction solution was exposed to visible light, samples were taken at predefined intervals. The temperature at which the reaction solution was collected was 25 °C, the transmembrane pressure was fixed at 1.65 MPa, and the feed water contained 0.5 g/L of humic acid. At 254 nm, the content of humic acid was determined with a Cary UV-2450 spectrophotometer.^61^. The rate of decomposition of humic acid is represented by the following equation (Eq. (7)) that illustrates the activity of photocatalysis. Where the average humic acid concentrations are represented by C_0_, C, and the Humic Acid Degradation (η%)^62^:

7 $$\upeta =\frac{\text{C}0-\text{C}}{\text{C}0} \times 100{\% }$$

A Hanna HI83399 multi-parameter photometer was used to test the COD in the water samples, while an Adwa (AD 310) conductivity meter was utilized to measure the TDS. Turbidity was measured using a Turbidimeter Benchtop made in China. The membrane was retested in sunlight at 30 °C, and light irradiation was 250 KW.h/m^2^. 

### Measuring the quantity of NPs in the permeate resulting from the TiO_2_/MnO_2_ membrane

For each membrane, 50 mL of permeate was collected to quantify the amount of cerium emitted from the membranes during filtration. After the permeate sample was acid-digested and diluted with deionized water, it was subjected to an inductively coupled plasma (ICP-5100 OES) analysis to determine its TiO_2_ and MnO_2_ content^63^.

## Results and discussion

### NPs

#### XRD

**Fig. 2:**
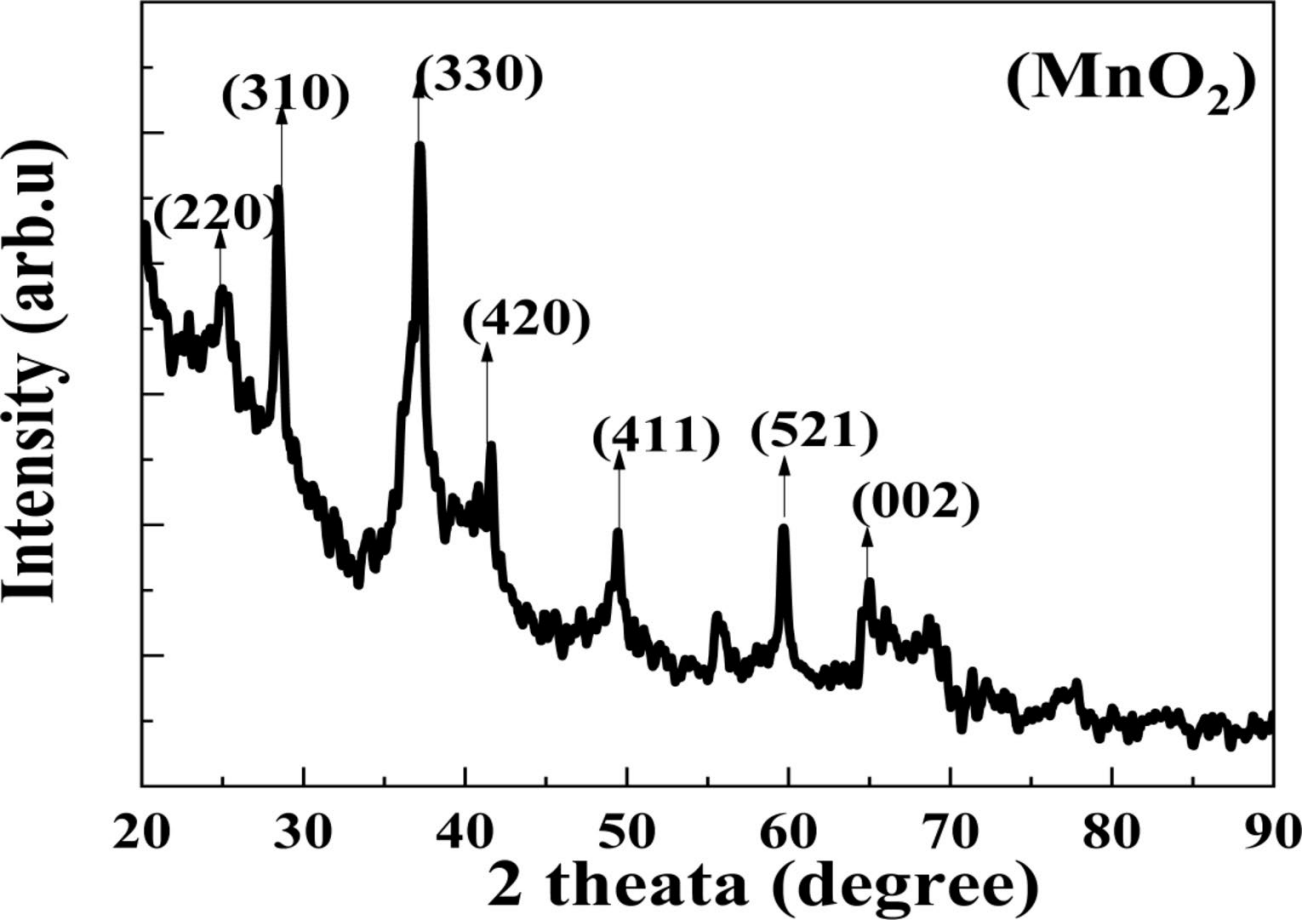
The XRD patterns of prepared MnO_2_ NPs.

**Fig. 3:**
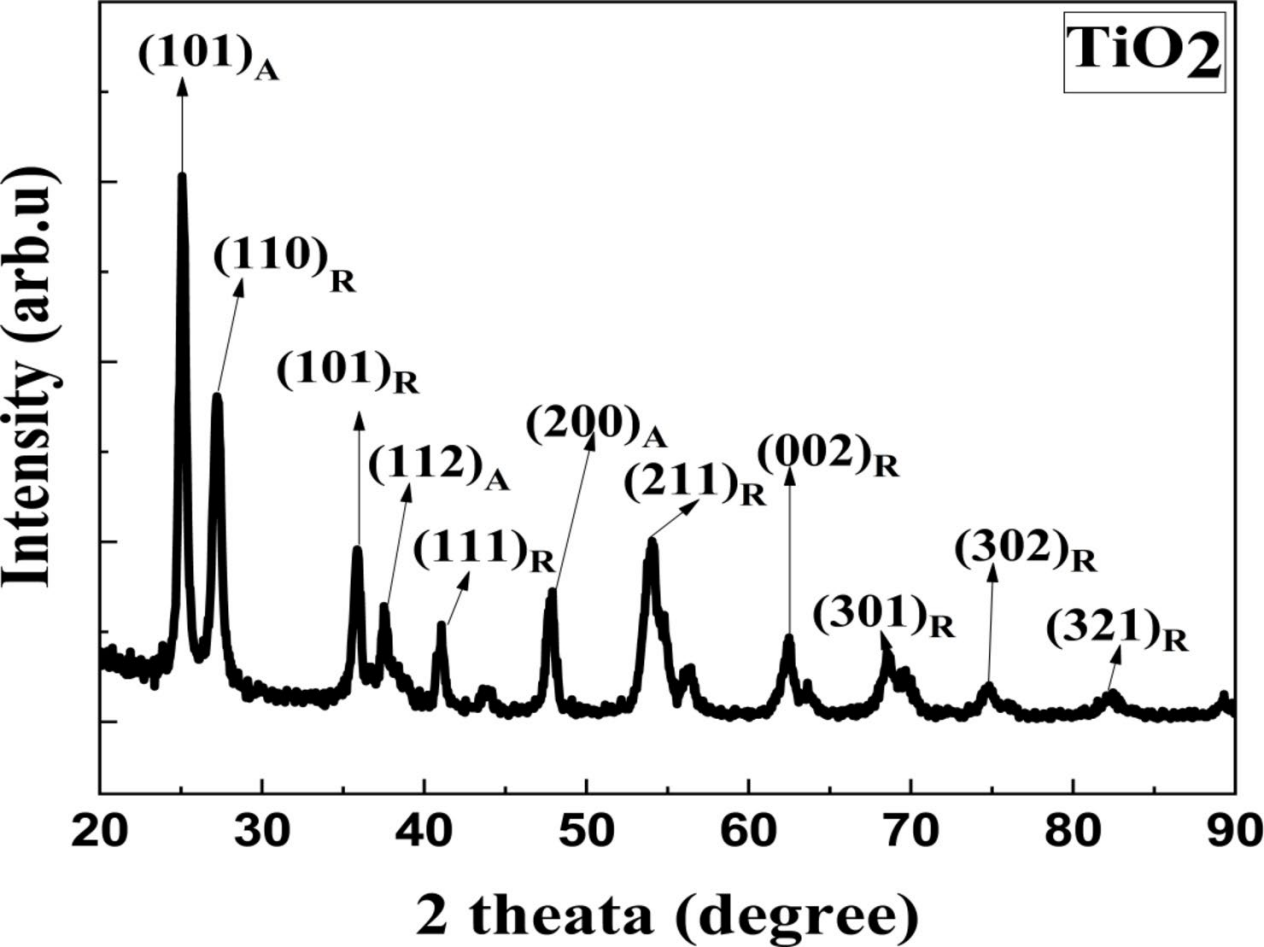
The XRD patterns of prepared TiO_2_ NPs.

The formed NPs’ crystal structure and the crystallite size were determined using the XRD spectrum (Figs. 2, and 3). The produced TiO_2_ NPs’ typical XRD configuration showed several distinct diffraction reflections. The presence of characteristic peaks at 2ɵ = (25.7, 39.2, 48.8, 63.1,69.9, and 76.4) for (101), (112), (200), (213), (116), and (215) crystal planes with an anatase phase and a tetragonal structure [File Card No: 04–014-0493]. Also, diffraction reflections appeared at 2ɵ = (27.5, 36.1, 39.3, 54.4, 56.7, 62.8, 64.2, 69.1, and 74.6) for (110), (101), (112), (200), (211), (220), (002), (310), (301), and (320) crystal planes exhibiting a rutile phase and a tetragonal structure [File Card No:04-012-7240]. Additionally, distinctive MnO_2_ peaks were found at 2ɵ = (25.12, 28.46, 37.16, 41.51, 49.31, 59.70, 65). For α-MnO_2_, JCPDS NO. 44-0141, (220), (310), (330), (420), (411), (521), and (002) crystal planes, correspondingly. The approved nanoparticles had their lattice constants and α-MnO_2_ index^64–67^. The relationship (Eq. 4) can be used to determine the crystallite size of the produced NPs based on the broadening of the peak in the XRD patterns. The produced NPs’ good crystallinity was confirmed by the XRD peaks’ strong diffraction reflections. The pattern showed no distinctive reflection associated with the contaminants. For TiO_2_, it was 24 nm, while for MnO_2_, it was 20 nm^68^. The mass fraction of the anatase phase to the rutile phase W_R_ was 0.06%. Despite the 450 °C calcination temperature, two phases anatase and rutile were seen in the TiO_2_ NPs. Because the rutile phase was impacted by the reaction time and the TiO_2_ NPs were prepared substantially more quickly when the reducing agent was the green extract, than when the chemical technique was used. Additionally, metal ions that were derived from plant extract throughout the TiO_2_ NPs manufacturing process have an impact on the rutile phase^56,69^. TiO_2_ and MnO_2_ NPs had dislocation densities of 1.7 and 0.9 × 10^15^ m^2^, respectively, and macrostrains of 0.0017 and 0.0009, respectively (Table 1). The produced NPs’ small dislocation densities suggest an elevated degree of crystallization^70^.

**Table 1:** The crystallite size (D), δ, and ε, of the prepared TiO_2_, and MnO_2_ NPs.

Nanoparticle	D (nm)	δ (× 10^15^) m^2^	ε
TiO_2_	24	1.7	0.0017
MnO_2_	20	0.9	0.0009

#### FTIR spectra

**Fig. 4:**
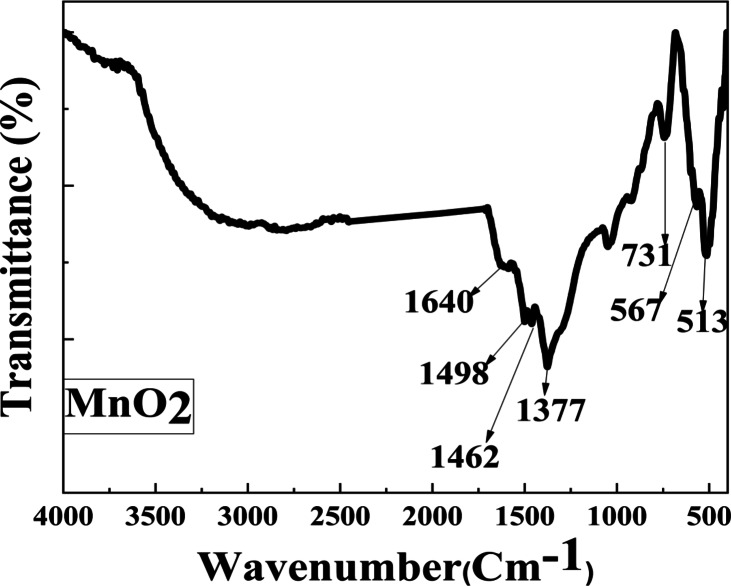
The FTIR spectra of the produced MnO_2_ NPs.

**Fig. 5:**
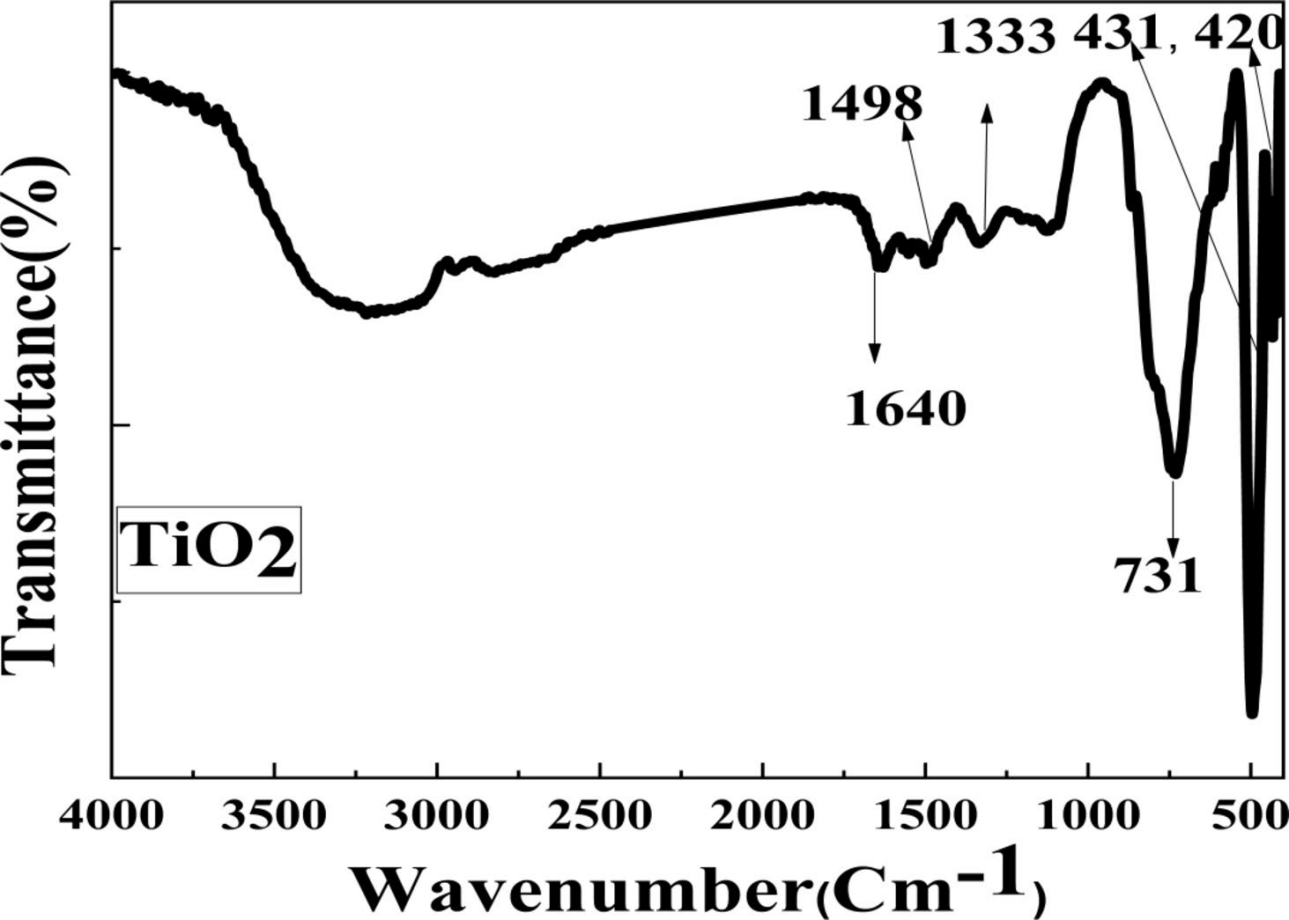
The FTIR spectra of the produced TiO_2_ NPs.

The FTIR of MnO_2_, and TiO_2_ NPs samples is displayed in Figs. 4 and 5. The Absorption band for TiO_2_ and MnO_2_ NPs is located at 1333 and 1377 cm^−1^, respectively, as a result of C and H’s combined stretching vibration. Furthermore, at 1462 cm^−1^, the O–H bending or C–C stretch peaks were seen. Conversely, a peak appears at 731, and 733 For TiO_2_, and MnO_2_ NPs respectively. The functional group O–H bending appeared at 1640 cm^−1^ for TiO_2_ and MnO_2_ NPs. A band for TiO_2_ NPs was found at 1498 cm^−1^, which is allied with the carbonyl (C = O) and C = C stretching groups. For MnO_2_ NPs, were observed at band 1498 cm^−1^. Peaks at 420, and 431 cm^−1^ show the existence of TiO_2_ NPs^71,72^ while bands at 567, and 513 cm^−1^ show MnO_2_.

### Membrane characterization:

#### FT-IR, and Raman spectra

**Fig. 6:**
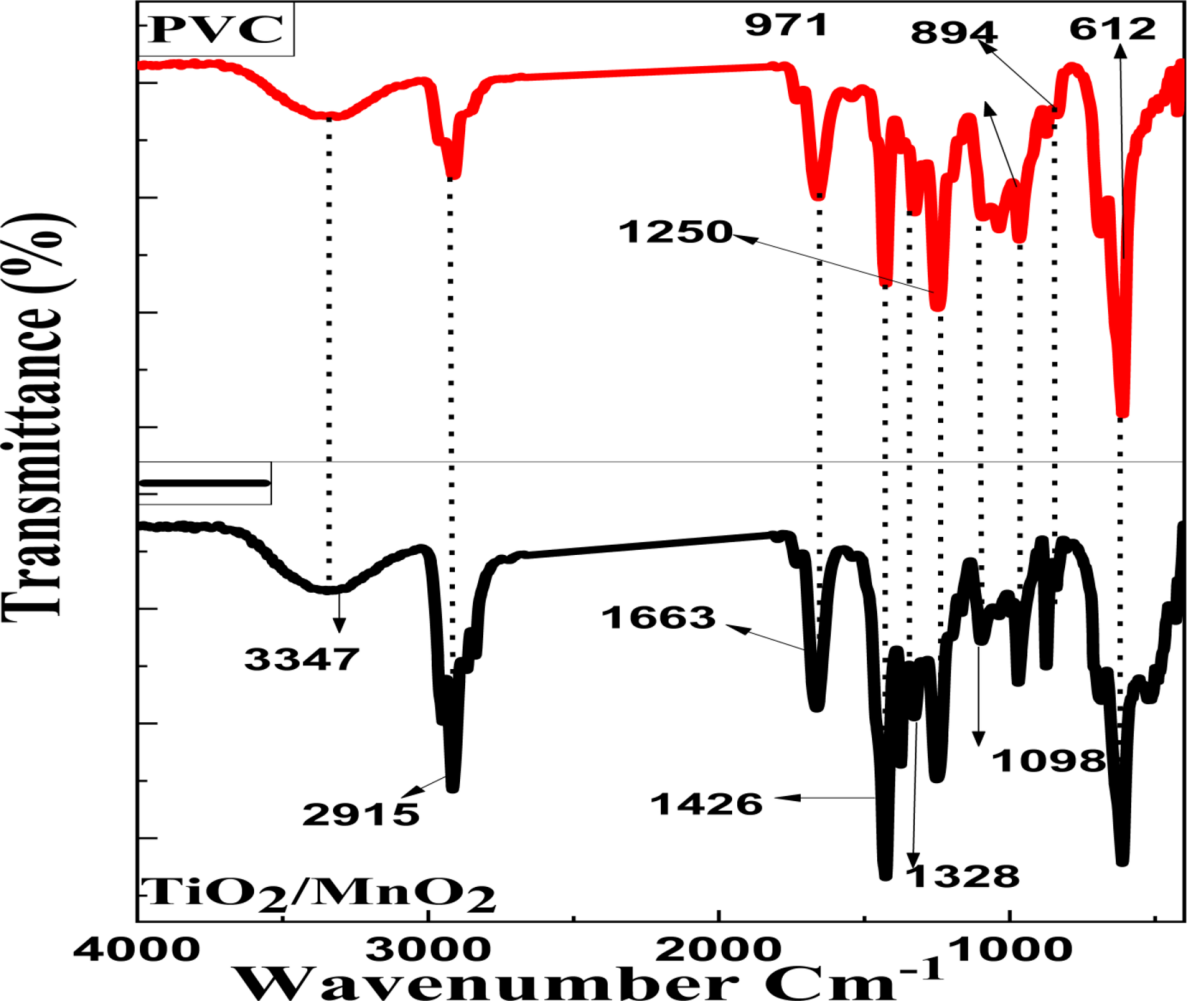
FTIR spectra for the prepared blank PVC, and TiO_2_/MnO_2_ membrane.

**Fig. 7:**
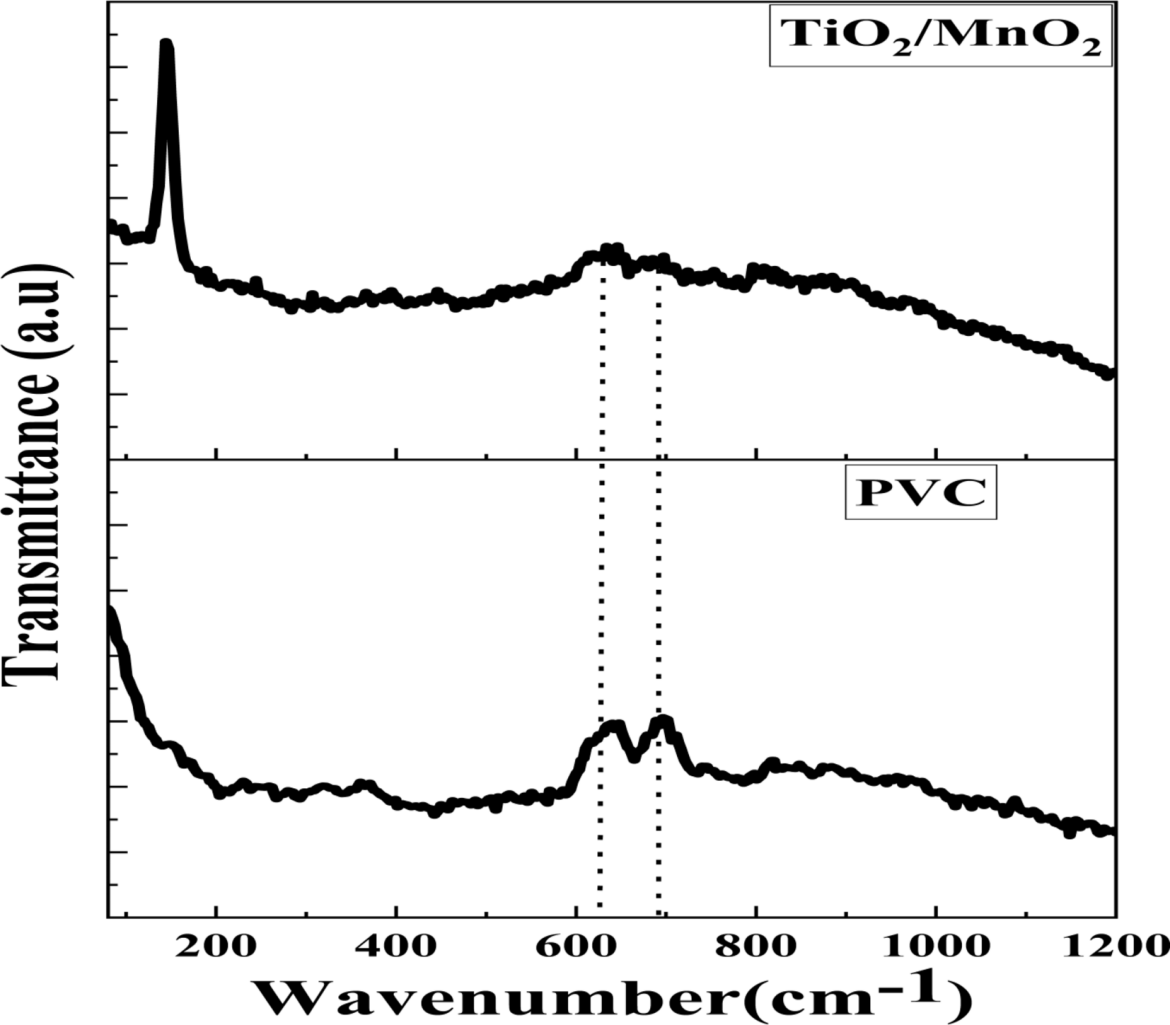
Ramman spectra for the prepared blank PVC, and TiO_2_/MnO_2_ membrane.

FT-IR was used to analyze the chemical structure of the nanocomposite PVC membrane. Figure 6 shows the incorporated NPs did not disturb the PVC polymer structure, as evidenced by the nanocomposite membranes’ display of all the distinctive bands of pure PVC, including bands and their assignments. Approximately 3347 cm^-1^ was found to be the representative stretching vibration of carboxylic groups and water hydroxyl (-OH). At 2915 cm^-1^, stretching vibrations of the C-H bond are observed. The stretched carbonyl (C = O) and C = C groups bands are at 1663 and 1426 cm^-1^ stretched, one-to-one. The absorption stretched band at 1328 cm^-1^ is the result of the combined stretching vibration of C-H, which is matched by the bands at 894, 1098, and 1347 cm^-1^. The stretching vibration of the C–Cl bond on the PVC also produced a band at 612 cm^-1^. The substitution of nucleophilic molecules of the PVC membrane, which led to a portion of the -Cl being exist, was the cause of the bands mentioned above. Several hydrophilic groups with validated FTIR spectra could be compatible with polymer chains. The dope solution becomes more hydrophilic with adding NPs, increasing the rate at which solvents and nonsolvent exchange during phase separation. Furthermore, there are numerous bending vibrations in the absorbent bands measuring 971 and 1250 cm^-1^, both outside and inside the C-H bonding plate. Additionally, Raman spectroscopy was used to examine the produced membrane Fig. 7. Two strong distinctive bands for PVC may be seen at 636 and 693 cm^-1^ because of the C–Cl stretching vibration^73,74^. An acute peak was detected at 144 cm^-1^, corresponding to the anatase phase of TiO_2_ NPs. The six distinct Raman active modes are revealed via factor group analysis (A1g + 2B1g + 3Eg). When Ohsaka examined the Raman spectra of a single anatase crystal, he discovered that the six permitted modes could be seen at 144 cm^-1^ (Eg), 197 cm^-1^ (Eg), 399 cm^-1^ (B1g), 513 cm^-1^ (Eg), as well as 519 cm^-1^ (B1g), and at and 639 cm^-1^ (Eg)^75–77^.

#### The morphology of the prepared membrane

**Fig. 8:**
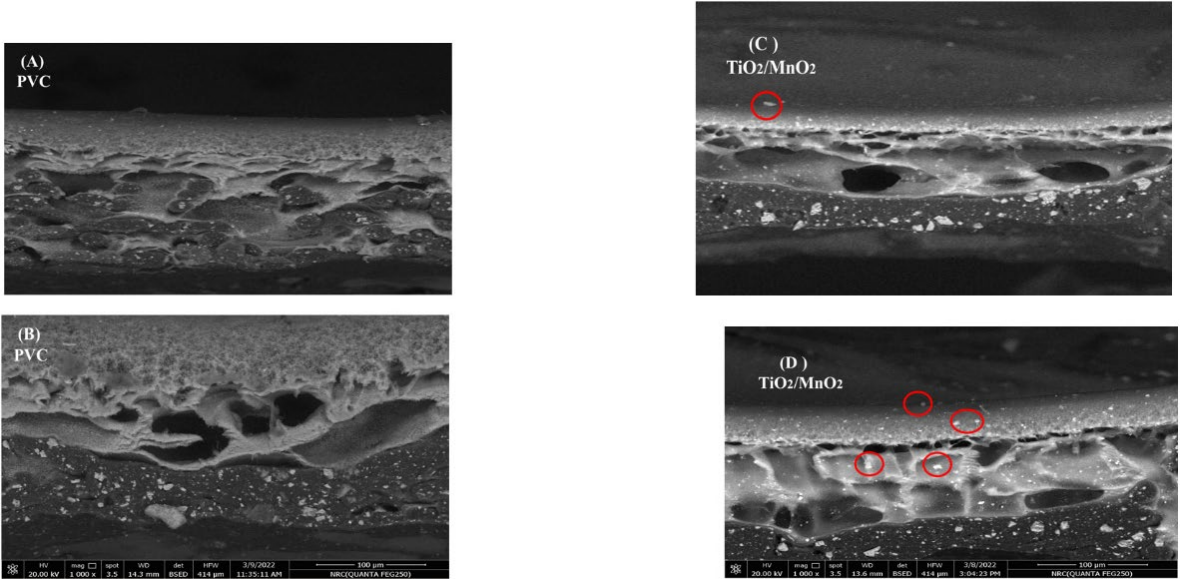
FESEM membranes morphology (**A, B**) for PVC, and (**C, D**) for TiO_2_/MnO_2_ membrane.

Figures 8 (A, B, C, and D) show cross-sectional FESEM pictures of the PVC blank and TiO_2_/MnO_2_ membranes created. It has an asymmetrical construction that A thick skin layer and flexible, finger-like sublayers make up the asymmetrical porous structure of all membranes. The layer above controls the absorption and rejection of solutions, whereas the membrane’s sub-layer protects and reinforces the membrane from damage. This structure is formed by fast mass transfer between the solvent and the non-solvent because of non-solvent phase inversion. It is well known that the dense top layer enhances separation efficiency by lowering permeability. The addition of NPs to the PVC casting solution alters the kinetic and thermodynamic properties of the system. The lack of NP accumulation suggests that TiO_2_ and MnO_2_ NPs are distributed uniformly over the injection solution and the membrane polymer. Furthermore, the lack of surface fractures on the membrane indicated that the incorporation of NPs had no detrimental effects on their strength and did not induce brittleness in the membranes^78^. NPs added to the polymeric matrix operate as hydrophilic fillers, facilitating diffusion transfer between solvent and non-solvent throughout phase inversion, leading to create a finger-like sublayer^57^. The polymeric solution is cast onto the polypropylene support everywhere it permeates the support, resulting in a spongy bottom structure. The membrane is stretched by the macro voids, often known as finger-like holes. The dope solution’s growing viscosity has an impact On the Kinetics of the Phase Inversion Mechanism, this causes different detrimental effects, involving a decrease in the rate at which membranes build up and the porosity formation. That resemble sponges among the macro void pores, plus the growth in the overall number of macro void pores^79^. The red circles around the white sites in the membranes indicate the presence of the nanoparticles, which are the controllers of the photodegradation of contaminants.

**Fig. 9:**
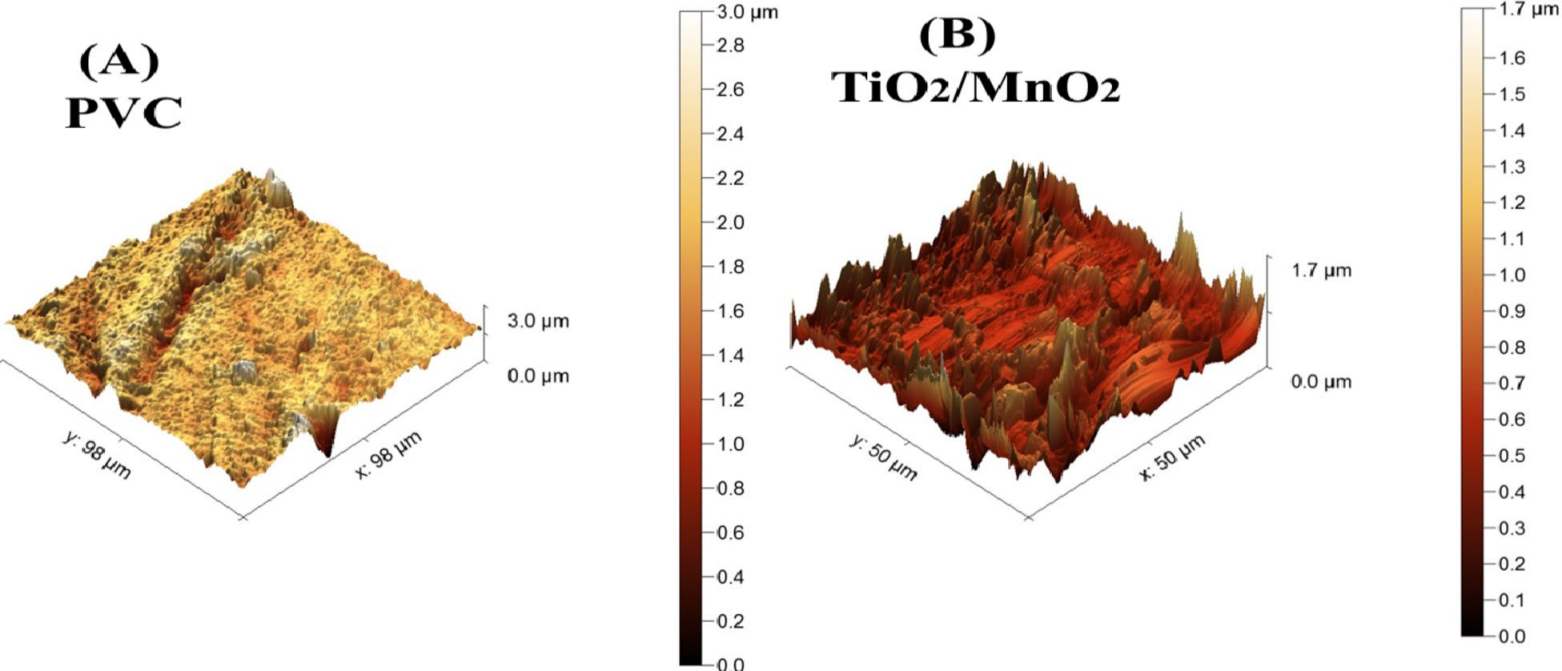
AFM membranes morphology (**A**) for PVC and (**B**) for TiO_2_/MnO_2_ membrane.

As shown in Fig. 9(A, and B), the rough microstructures on the membrane’s surface were observed. Peaks and valleys, or pores and brightness, respectively, indicate the image’s darkest and brightest regions. as indicated by the AFM data in Table 2. The produced membranes have roughness values of 72.4 ± 0.2 and 5.8 ± 0.02 nm for blank and TiO_2_/MnO_2_. TiO_2_/MnO_2_ has lower Ra, suggesting that decreasing surface roughness reduces the possibility of contaminants building up the membrane’s upper roughs. The behavior of the polymeric membranes’ anti-fouling is enhanced as a result: growing surface roughness and increased surface area cause the membrane flow to increase^80^. This rising tendency could be mostly attributed to the NPs’ capacity for accumulating on the surface of the membrane when there is a high concentration of nanofiller presence. It is thought that surface roughness has a major impact on membrane resistance to fouling, with smoother membranes performing improved in this area. Among the factors that could affect the surface attributes are the formation of electrostatic interactions between polymer chains, bending, wrinkling, chain density, and surface area variations. The important smooth surface here is essential to reducing the chance of fouling. The more inconsistent the topography of the membrane, the greater the probability of fouling. This increasing trend may be mostly When there is a lot of nanofiller, the NPs prefer to concentrate on the membrane surface. Surface roughness is assumed to have a major impact on membranes’ resistance to fouling; membranes having a smoother surface exhibit higher performance in this context^81^. To reduce the possibility of fouling, this smooth surface is essential. The more irregular the topography of the membrane, the greater the likelihood of fouling.

**Table 2:** Contact angle porosity, roughness, and thickness for (blank, and TiO_2_/MnO_2_) prepared membranes.

Membrane	Contact angle degree	Porosity (%)	Ra (nm)	Thickness
Blank (PVC)	69 ± 1.9	59 ± 0.3	72.4 ± 0.2	200 µm
TiO_2_/MnO_2_	68 ± 0.4	83 ± 0.5	5.8 ± 0.02	200 µm

#### The porosity and the contact angle

TiO_2_/MnO_2_ membrane has a total porosity of 83 ± 0.5%. The porosity capacities show that it has good porosity. This suggests that the NPs enhance the membrane’s properties by improving the interior structure’s porosity, which may raise the membranes’ lateral flow rates. It has a contact angle of 68° ± 0.4 (Table 2). According to the rule, When the contact angle is < 90⁰, a material is hydrophilic, and hydrophobic and has little wetting if it is ˃90⁰. The models became more suitable for water flux testing as the contact angle decreased below 90⁰, The water contact angle results showed that the TiO_2_/MnO_2_ modification of the PVC membrane decreased the contact angle from 69 ±1.9° for the neat PVC membrane to 68 ± 0.4° for TiO_2_/MnO_2_, indicating an improvement in the hydrophilicity of the membrane. Indicating that the attempt was successful the same behavior showed in later research^82,83^. The TiO_2_/MnO_2_ membranes had a greater bulk porosity of 83 ± 0.5%, mostly because of the creation of "fine-sized" macro-voids, compared to the blank PVC membrane’s 59 ± 0.5% bulk porosity. Increasing the quantity of NPs enhances their hydrophilicity since NPs are naturally hydrophilic as shown in^84,85^.

#### XPS

**Fig. 10:**
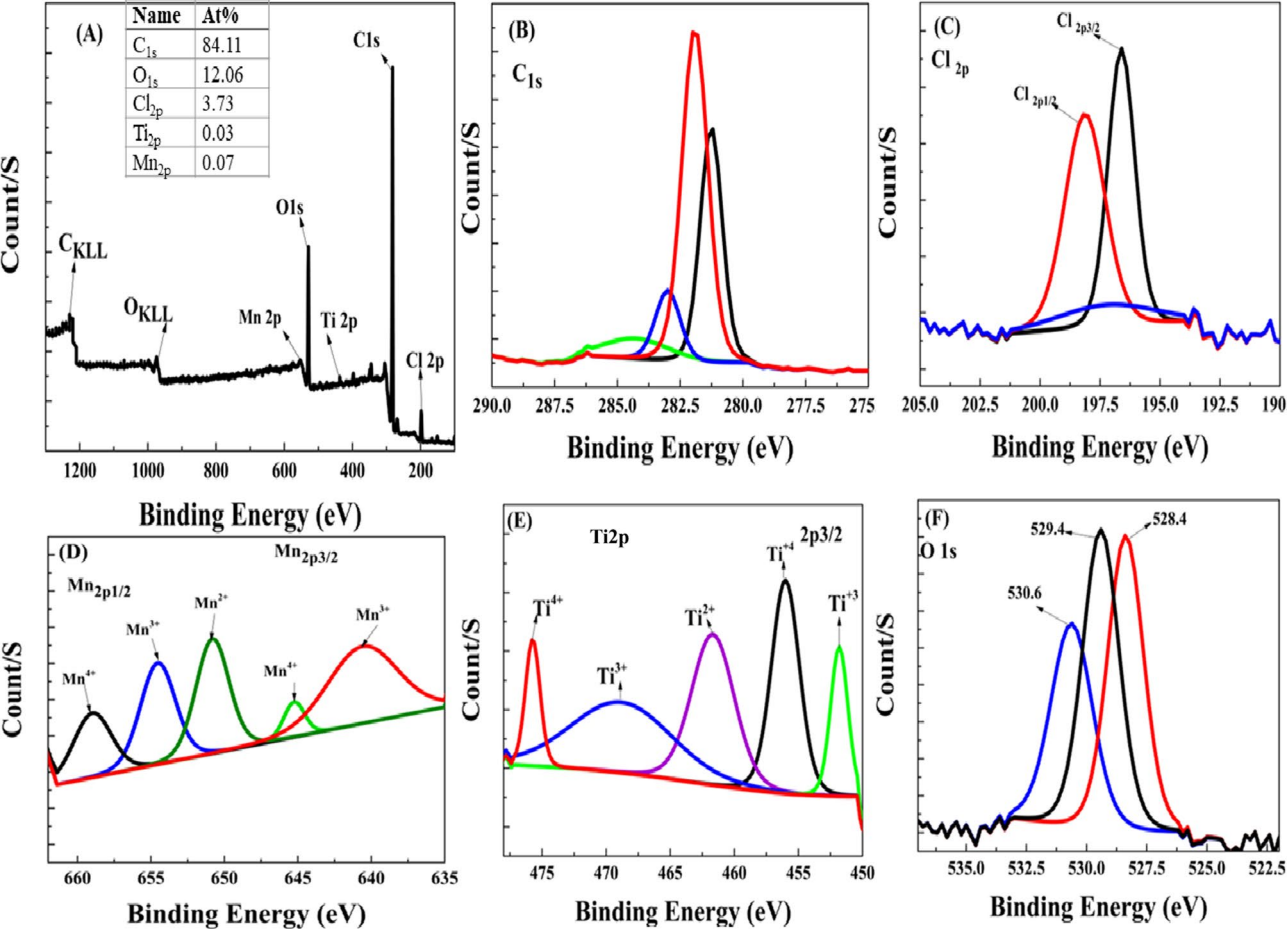
X-ray photoelectron spectroscopy (XPS) pattern of TiO_2_/MnO_2_ Membrane Full spectrum with elemental concentration (**A**), Peak and high-resolution spectrum of C1s spectrum (**B**), of Cl2p (**C**), Mn2p (**D**) , Ti2p (**E**) and O1s (**F**).

The generated TiO_2_/MnO_2_ composite membrane’s surface chemical compositions were assessed using XPS, and the results are displayed in Fig. 10. The sample contains just Ti, Cl, C, O, and Mn, in good agreement with the XRD result. At 1224, 976, 554, 530, 347, 302, 284, 270, and 201 eV, the main peaks emerged. C_KLL_, O_KLL_, and Mn_2p_ are associated with the peaks located at 1224, 976, and 554, respectively. Cl_2p_, Cl_2s_, C_1s_, and O_1s_ emissions are responsible for the four main emission peaks at 201, 270, 284, and 530 eV, respectively. The elemental concentration (at.%) values also confirmed that (Fig. 10 (A)). Since PVC membranes don’t contain oxygen, the O_1s_ peak near the membrane surface is thought to be representative of the PVC component. Surface oxidation by ambient oxygen is linked to the emergence of a little O_1s_ peak in the pure PVC membrane^86–89^. We think that all of the produced membranes experience this surface oxidation. Additionally, the TiO_2_/MnO_2_ Membranes’ oxygen O_1s_ deconvolution primarily displays three peaks in Fig. 10 F).

Lattice oxygen on TiO_2_ is responsible for the first one, which occurs at about 530.6 eV; lattice oxygen of metal oxide (Mn–O–Mn bonds) is responsible for the second one, which occurs at 529.4 eV^90,91^. In the produced membranes, there is an extra peak at about 528.4 eV that is associated with metal-bound oxygen. Because they help supply active oxygen species during the process, the OH groups are important^92^. Since the O lattice of TiO_2_ and MnO_2_ is responsible for the O_1s_ at 530.62, 529.4, and 528.38, as well as the oxygen vacancies^93,94^, the Ti–O-Ti bonding peak shifts to a lower binding energy at 529.7 eV, indicating the presence of Ti^3+^. The presence of Ti^3+^ and surface modification with a hydroxyl group are advantageous for pseudocapacitive activity^95,96^.

As shown by the Ti_2p_ XPS spectra shown in Fig. 10 E), which include four peaks related to Ti_2p1/2_ (468.8 eV), Ti_2p1/2_ (456.01 eV), and Ti_2p3/2_ (461.7 eV) and Ti_2p1/2_ (475.7 eV) correspondingly. Makes it clear that the Ti^4+^ peak is in the doublet state, and that the accompanying peaks are associated with Ti_2p3/2_ and Ti_2p1/2_, which is in line with previous reports. Furthermore, a wide peak that corresponds to Ti^3+^ (2p1/2) is present. The region under the Ti^4+^ peak directly indicates an increase in the amount of oxygen vacancies. The creation of oxygen vacancies during the preparation is shown by the decrease of Ti^4+^.^97^ Ti is present in the Ti^4+^ state, Ti^3+^. To elicit the Mn species, the deconvolution was carried out concerning to manganese 2p spin–orbit doublet peaks, where five peaks originating from Mn^2+^, Mn^3+^, and Mn^4+^ can be differentiated Fig. 10 D). confirming a complicated multiplet electronic structure that primarily contains (Mn^4+^, Mn^3+^, and Mn^2+^) Mn_2p1/2_ at 658.98, 654.54, 650.81, and (Mn^4+^, Mn^3+^,) Mn_2p3/2_ at 645.25, and 640.59^98^. The primary peak in the C_1s_ XPS spectra was found at 284.26 eV binding energy, which matched the C–C coordination^99^ Fig. 10 B) . Cl_2p1/2_, and Cl_2p3/2_ were observed 198.13, and 196.61 Fig. 10 C).

#### Pore size and pore radius distribution

**Fig. 11:**
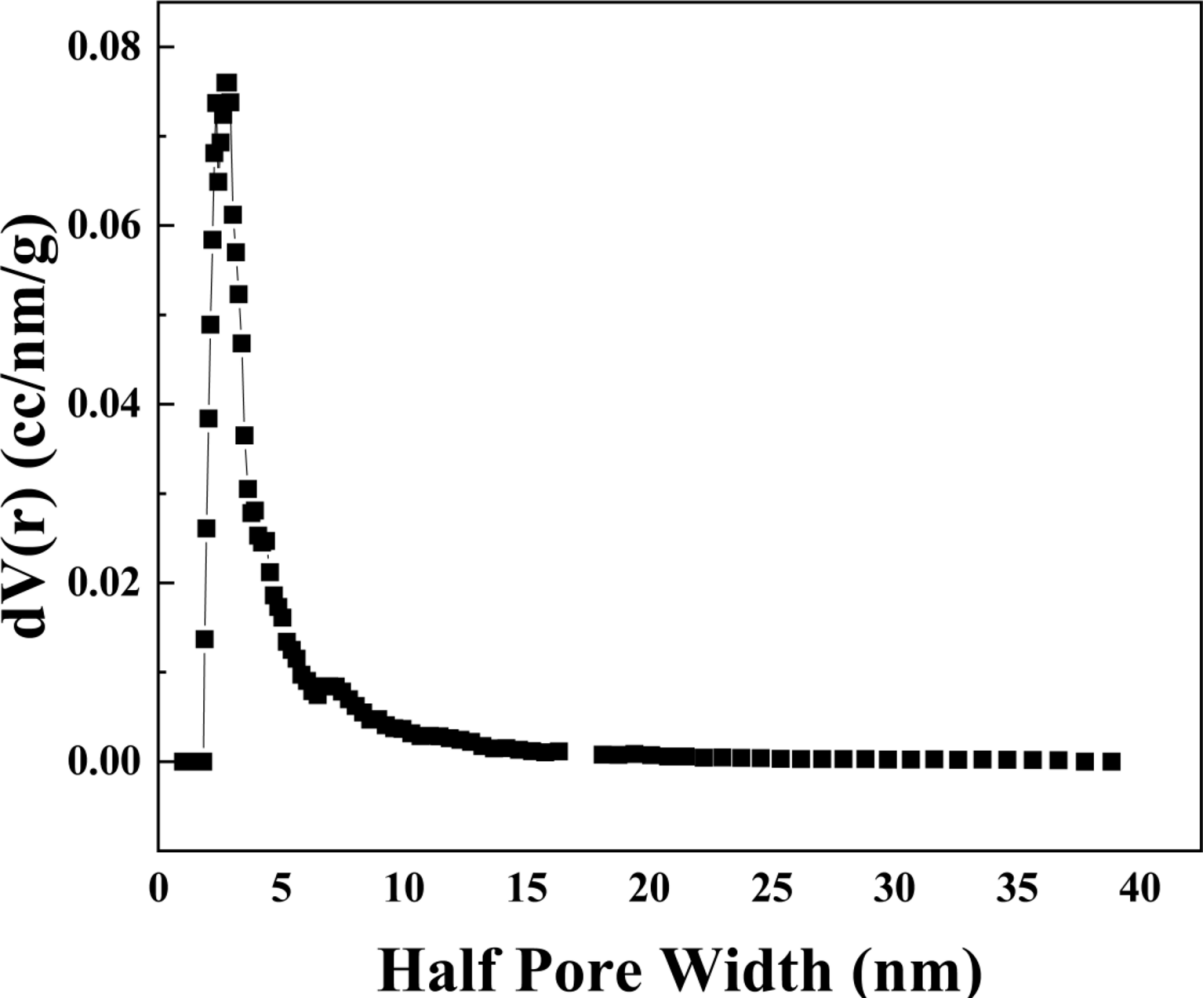
The Pore radius distribution of the Barrett (BJH curve) of the synthesized TiO_2_/MnO_2_ membrane.

**Fig. 12:**
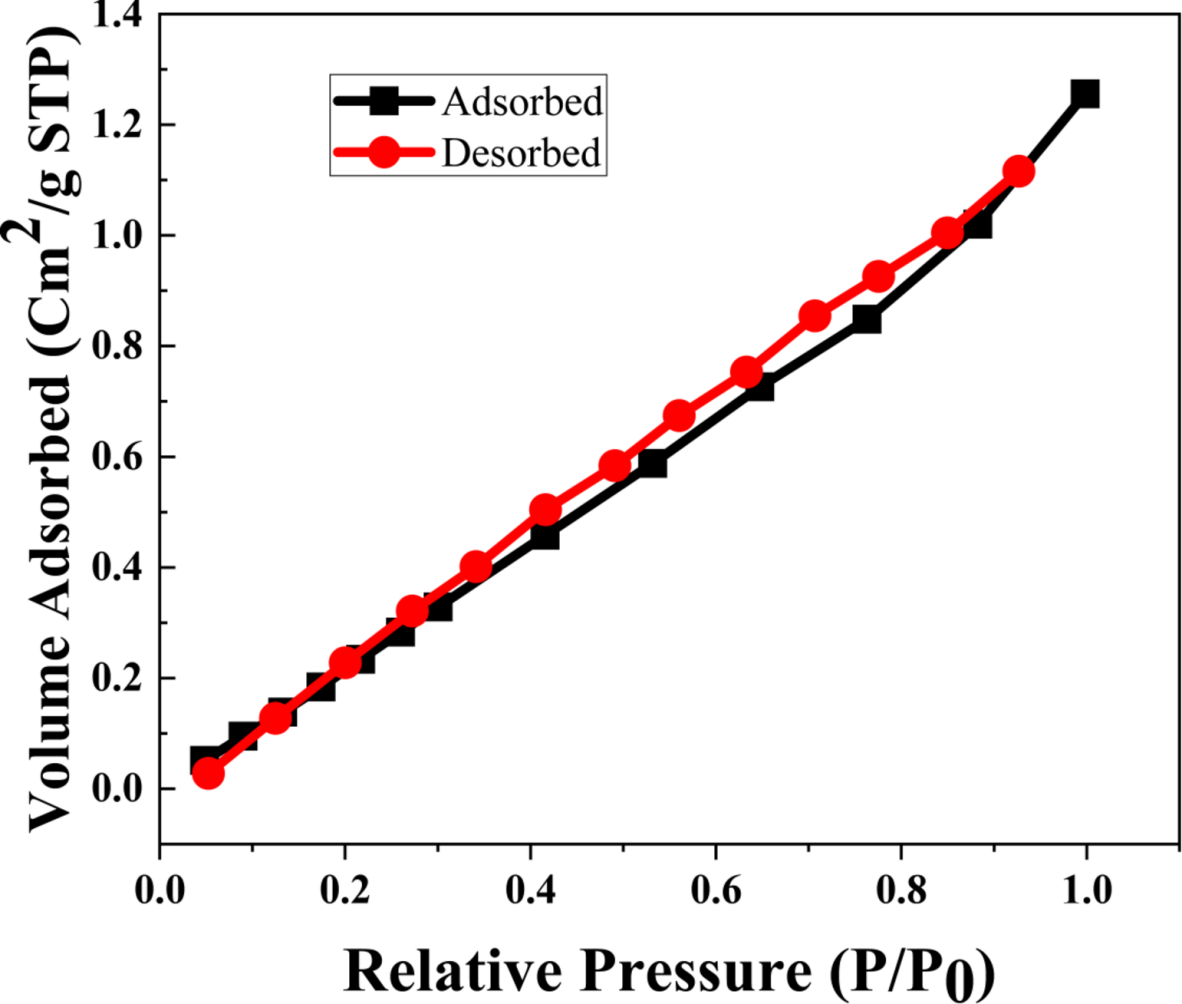
Nitrogen adsorption–desorption isotherm measured of the synthesized TiO_2_/MnO_2_ membrane.

To determine the sample’s surface area, pore diameter, and pore volume, the BET analysis was carried out. Figure 11 shows the Barrett–Joyner–Halenda (BJH) plot and the N_2_ adsorption–desorption isotherm of all produced samples. The adsorption/desorption curves of every constructed TiO_2_/MnO_2_ membrane exhibit a hysteresis feature typical of mesoporous materials^100^, as shown in Fig. 12. The International Union of Pure and Applied Chemistry (IUPAC) has classed the (Ads/Des)-isotherm as IV including an H_3_ hysteresis loop (IUPAC classification^101^). The (Ads/Des)-isotherm is classified as IV involving an H_3 _hysteresis loop. The behavior of adsorption of N_2_ gas molecules depends on the relative pressure, such that, in regions of lower pressure, the formation of a monolayer is followed by the formation of multilayers of the adsorbed molecules in the regions of higher pressure. The total surface area obtained using the BET method, Pore radius, and Pore volume are shown in Table (2). the measured pore size and pore volume which signifies TiO_2_/MnO_2_ Membrane as a mesoporous material. The porous materials have been classified into three categories by the International Union of Pure and Applied Chemistry (IUPAC) based on their porous sizes and diameter (d): microporosity (d < 2 nm), mesoporous (2 nm < d < 50 nm), and microporous (d > 50 nm)^102,103^. The BET surface areas increased with increasing N_2_ adsorption–desorption isotherms (a), pore size distributions (b) of the ZnO, and specific surface areas of the samples (c) formed under different conditions. Which indicates the sample has a peak below 10 nm and an average pore radius of 1.93 nm the surface area is Surface Area 90.48 m^2^/g.

### Wastewater treatment

#### Humic acid

**Fig. 13:**
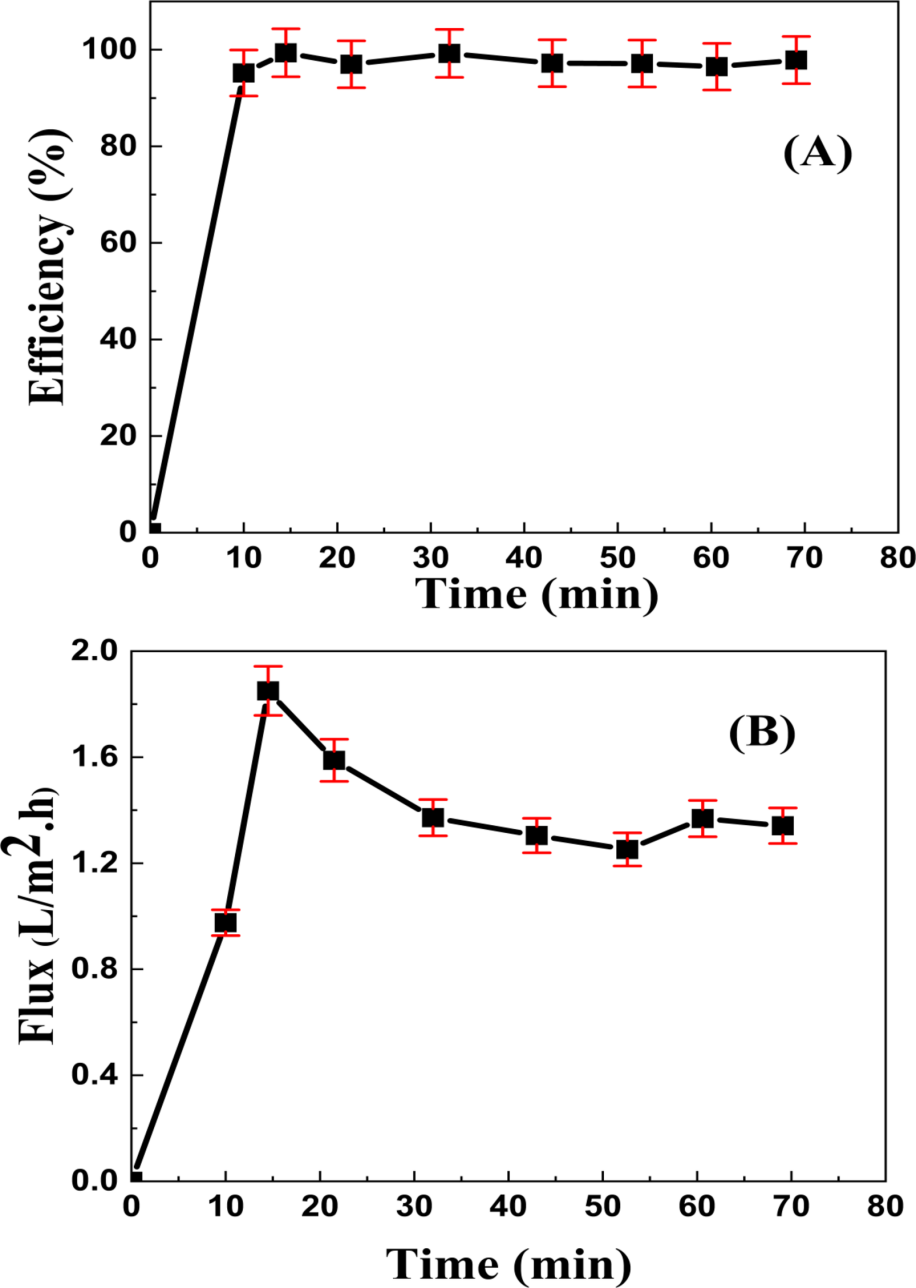
The elimination efficiency (**A**), and water flux (**B**) for the prepared membrane when sunlight is present.

**Fig. 14:**
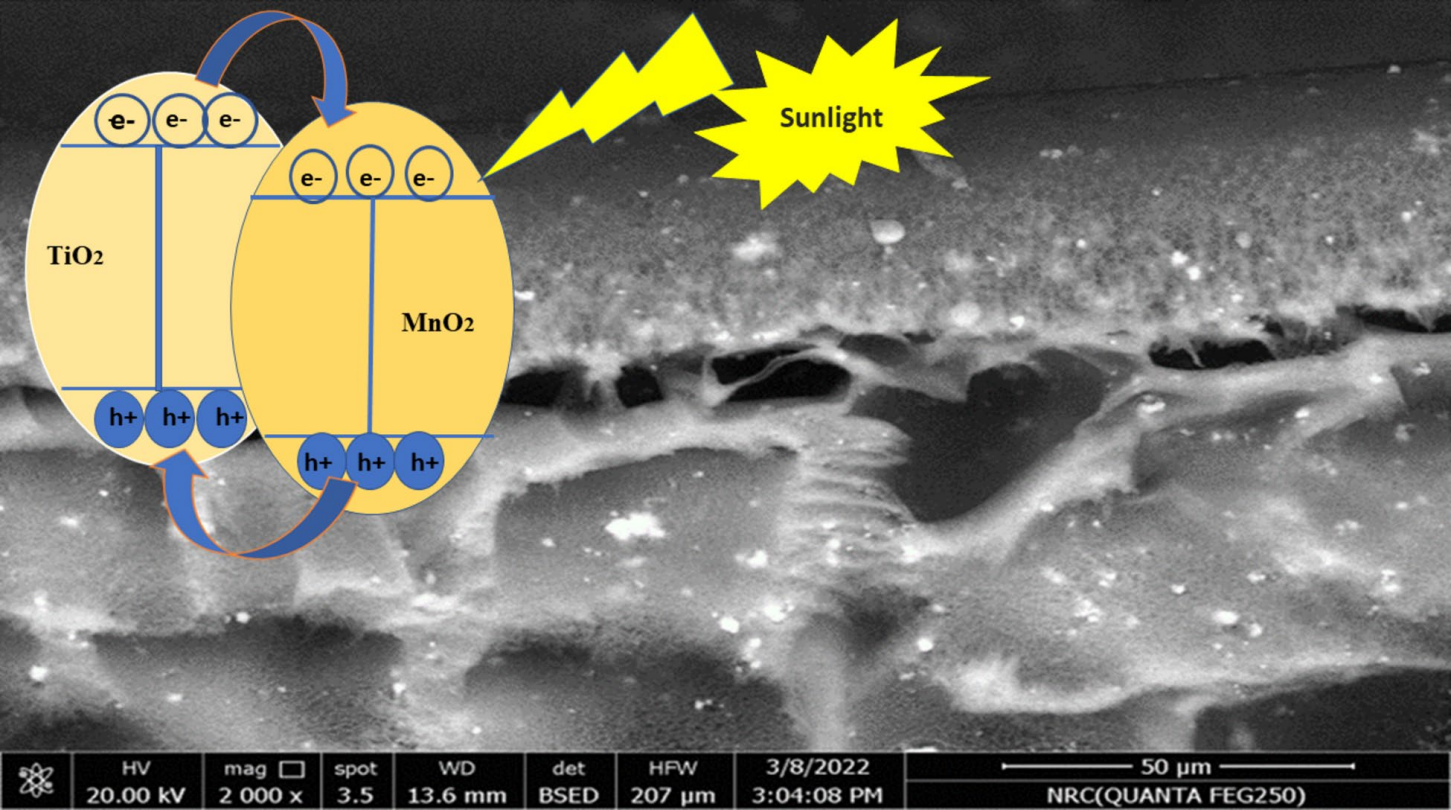
The photocatalytic mechanism inside the TiO_2_/MnO_2_ membrane.

After 70 min in the sun (With a temperature of 30 °C and a light intensity of 250 KW.h/m^2^), the membrane rejection rate and the water flux were examined; their values are shown in Fig. 13). The membrane removal efficiency (Rejection) is 97.5 ± 0.5%, and its clean water flux equals 1.5 ± 0.01 L/(m^2^.h). The following reaction demonstrates how organic compounds in organic wastewater can be photocatalytically degraded in the existence of a TiO_2_/MnO_2_ when exposed to sun radiation. However, in this instance of photocatalytic membranes, the photocatalytic elimination of pollutants goes hand in hand with the filtering mechanism that gets rid of bigger particles. Metal oxide nanoparticles’ mechanism was explained in one of our published papers. Oxygen and water were grabbed by excited state electrons when exposed to visible light, forming the superoxidants O_2_• − and OH•, which then broke down organic pollutants to release carbon dioxide and water. The hydroxyl radicals that the photocatalyst creates in response to light exposure eliminate the contaminants that have been retained. OH^•^ and O_2_^•^ − radicals are the primary oxidizing species, per previous studies^56,57^. Furthermore, when the pollutants come into contact with the membrane surface doped with NPs, they undergo simultaneous reduction and oxidation due to an unoccupied electron–hole on the photocatalyst. This stops cake layers from forming on the membrane’s surface, which could hinder membrane permeability and photocatalytic activity. The photocatalytic breakdown of organic materials in organic wastewater, which is aided by a TiO_2_/MnO_2_ nanocomposite and solar light, is demonstrated by the reaction that follows. As seen in Process (eq: 8–13), the reduction of the superoxide anion (O^•^_2_¯) by the electron in the conduction band CB (e¯_CB_) results in the creation of the hydroxyl radical (OH⋅), the main catalyst for the photodegradation of organic materials. The organic contaminants in the wastewater are then broken down by hydroxyl radicals (OH^•^), which cause them to mineralize into CO_2_ and H_2_O. It has been suggested that through the combination of hydrogen bonds and van der Waals forces with water molecules, the strong polarity hydroxyl radicals generated during the photocatalytic reaction could enhance the flow. On the other hand, as seen in Fig. 14 the little decrease in flow was ascribed to moderate membrane fouling, in which humic acid molecules stuck to the membrane’s surface and pores, creating narrow passageways and preventing water from passing through the membrane. Photoexcited electrons migrate from the CB of TiO_2_ to the CB levels of MnO_2_ when the TiO_2_/MnO_2_ nanocomposite gets exposed to sunlight, while holes migrate between the VB levels of TiO_2_ and MnO_2_ NPs. Thus, the lifetime of the charge carriers can be extended by increasing the efficiency of interfacial charge transfer to adsorbed molecules^58,79,104^.

Nevertheless, the reason for the flux decrease was tiny membrane fouling, where humic acid molecules stuck to the membrane’s pores and surface, which reduced the flow and made Water find it difficult to flow through^105,106^.

8 $${\text{MO}}_{{2}} + {\text{ h}}\upsilon \, \to {\text{ MO}}_{{2}} ({\text{e}}^{ - }_{{{\text{CB}}}} + {\text{h}}^{ + }_{{{\text{VB}}}} ) \, ;{\text{ M}}:{\text{ Mn}},{\text{ Ti }}\left( {{\text{Photoexcitation}}} \right)$$

9 $${\text{h}}^{ + }_{{{\text{VB}}}} + {\text{ H}}_{2} {\text{O }} \to {\text{ H }} + {\text{ OH}}^{ \bullet }$$

10 $${\text{h}}^{ + }_{{{\text{VB}}}} + {\text{ OH}} - \, \to {\text{ OH}}^{ \bullet }$$

11 $${\text{e}}^{ - }_{{{\text{CB}}}} + {\text{ O}}_{2} \to {\text{ O}}_{2}^{ \bullet \, - } \left( {\text{Reduction in CB}} \right)$$

12 $${\text{O}}_{2}^{ \bullet \, - } + {\text{ H}}^{ \bullet }_{2} {\text{O }} \to \, 2{\text{OH}}^{ \bullet }_{2} \left( {\text{Radical generation in CB}} \right)$$

13 $${\text{OH}}^{ \bullet } + {\text{ Organic pollutants }} \to {\text{ CO}}_{2} + {\text{ H}}_{2} {\text{O }}\left( {{\text{Decomposition}}} \right)$$

#### Pulp and paper wastewater treatment

Moreover, industrial effluent (sewage from pulp and paper factories in upper Egypt) was treated using the produced membrane. Major causes of pollution during the different steps of the paper-making steps include the following: the processing of raw ingredients; pulping; washing the pulp; screening; washing; bleaching; paper machine operations; and coating operations. Absorbance, TDS, high organic material, and severe COD and Turbidity values are characteristics of the wastes. The pulp and paper industry’s effluents contain significant pollutants, that are generally regarded as being excessive levels of suspended solids and organic materials. One of the methods used to make paper is pulping, particularly when done chemically. This method produces effluent with a high concentration of organic material, suspended particles, wood debris, and soluble wood materials^14,107^ Table (3).

### (i) Lambda maximum

**Fig. 15:**
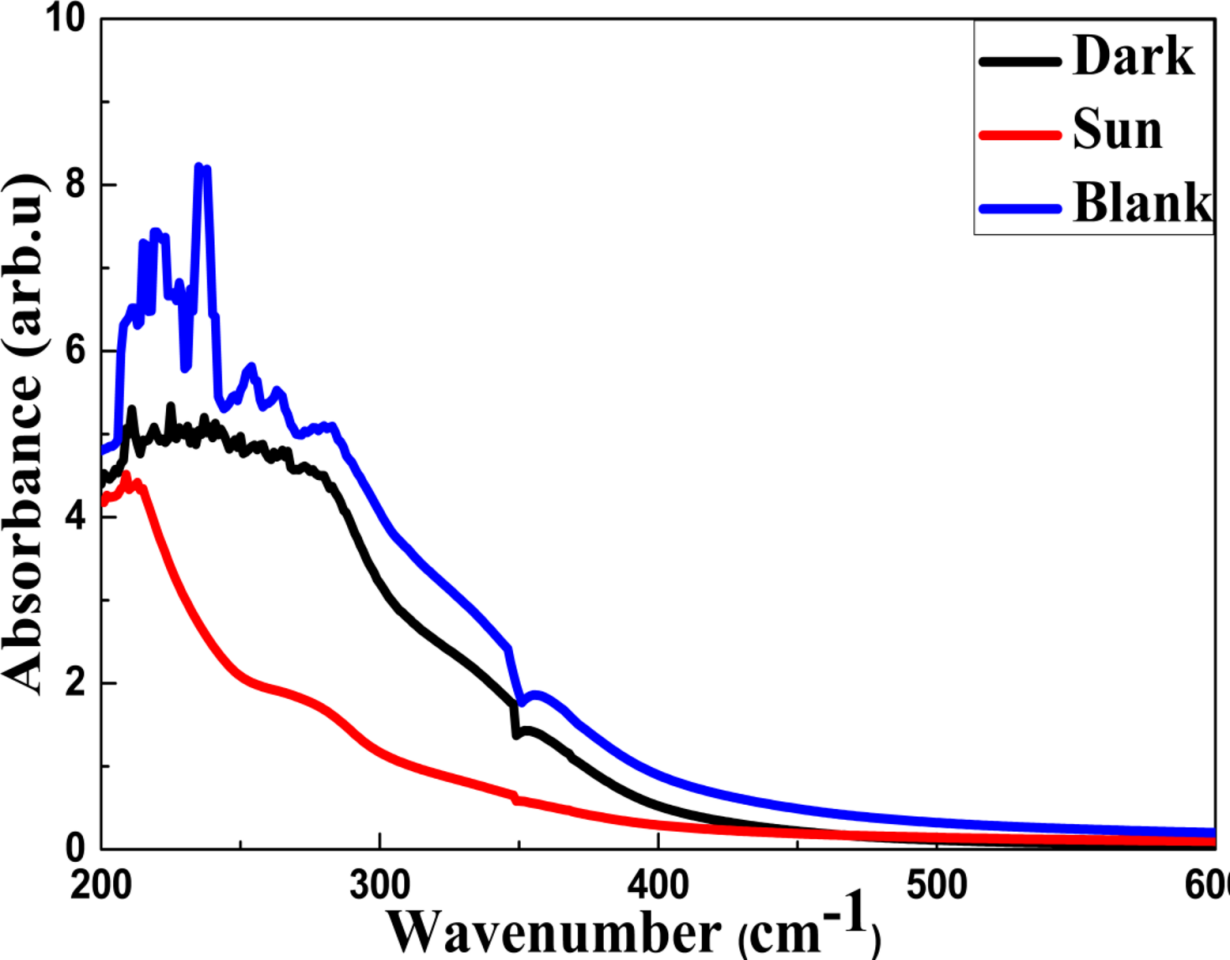
Absorbance values before and after treatment using TiO_2_/MnO_2_ membrane.

The Lambda maximum value of each wastewater sample was ascertained using a UV–vis spectrophotometer. The current study determined that 282 nm was the ideal wavelength for the most color reduction. The maximum absorbance removal value of 5.15 a.u). is also displayed along the y-axis in Fig. 15. In the current investigation, the absorbance and, finally, the color removal effectiveness were measured using this ideal and accurate wavelength. The color of the wastewater sample is indicated by the absorbance. Equation (14), which measures the percentage of color loss from wastewater samples, was utilized.

14 $$\upeta =\frac{\text{A}0-\text{A}}{\text{A}0} \times 100{\%}$$

### (ii) Determination of TDS

A liquid’s total dissolved solid (TDS) is a measurement of all the organic and inorganic components present, mostly consisting of salts, minerals, and organic matter. TDS is a helpful metric for assessing the general water quality. The effluent samples in the current investigation had the greatest TDS (1630 ± 1 ppm). The TDS levels in the wastewater discharged from each processing unit exceed the 500 ppm (500 mg/L) WHO maximum allowable limit for wastewater dumping into surface water^108^. Elevated levels of dissolved solids may potentially have technical implications. When wastewater with increased TDS is discharged into bodies of water, it can raise the salinity of the water, making it unsuitable for drinking and irrigation. As shown in Table 3 TDS was decreased from 1630 ± 2 to 1200 ± 2 when TiO_2_/MnO_2_ membrane was used in dark because of the filtration of salts, minerals, and organic matter. A great decrease in TDS ( it became 452 ± 1) was observed when the membrane was exposed to sunlight as a result of the photodegradation of most organic pollutants. Most pollutants were destroyed by NPS that blended on the surface of the membrane in the presence of sunlight.

**Table 3:** Absorbance, TDS, COD, and turbidity values before and after treatment using TiO_2_/MnO_2_ membrane.

Parameter	Before	After			
		Dark		Sun	
COD (mg/L)	1500 ± 5	1015 ± 1	32 ± 0.1(%)	247 ± 1	84 ± 0.4(%)
TDS	1630 ± 2	1200 ± 2	26 ± 0.2 (%)	452 ± 1	72 ± 0.2(%)
Turbidity (NTU)	278 ± 2	5 ± 0.04	98 ± 0.8(%)	25 ± 0.2	91 ± 0.8(%)
ABS m^-1^ (282 Cm)	5.15 ± 0.2	4.35 ± 0.1	15.5 ± 0.2 (%)	1.64 ± 0.02	68.2 ± 1 (%)

### (iii) Determination of turbidity

Turbidity, a crucial feature of wastewater, is caused by soluble colored chemicals, insoluble materials, and plankton. It can be quantified as a decrease in transmitted light intensity or an increase in scattered light intensity. The average turbidity value of the effluents was 278 ± 1.6 NTU. Increased turbidity hinders light absorption in the water, which lowers photosynthesis and dissolved oxygen generation. One way to gauge the effectiveness of the unit’s coagulation-flocculation process is to look at the turbidity of the wastewater. The results of the turbidity measurement show that all processing unit effluents have noticeably higher turbidity, and the values are higher than the WHO-mandated regulation limits of 5 NTU. (preferably less than 1 NTU)^108^.

### (v) Determination of COD

One of the most important indicators of the pollutant load in effluents is chemical oxygen demand, or COD, which is the quantity of oxygen needed to decompose both organic and inorganic materials. The COD mean value for effluents was found to be between 1500 and 4.52 parts per million. They exceed the permissible limit of 350 mg/L^109^. This implied that effluents were hazardous, which might be caused by high chemical concentrations combined with organic materials that are resistant to biological processes^110^. As a result of the success of the prepared membrane in the photodegradation of all these organic materials, this great improvement in reducing the COD value occurred, making it a very suitable solution for reducing fouling processes.

### Determining the quantity of NPs in the permeate

The possibility for additives to seep out of such an inorganic-blended polymeric membrane during use is one potential disadvantage. Before using these membranes in the separation process, the leaching potential must be examined since the inorganic material may create secondary contamination of the permeate. NPs must not be released into the permeate during filtration to be used as additives successfully. It is confirmed that the majority of the NPs utilized to change the membrane are kept in the membrane during filtering when the amount of NPs in the permeate for the manufactured TiO_2_/MnO_2 _membranes in water is less than 1 μg/L. Therefore, there is very little secondary pollution of the treated water. Our findings also showed that the amount of NPs in the permeates was comparable to that in the feed water, indicating that the membranes can be used for filtration without risk.

As shown in Table 4, the overall performance of methods used in pulp and paper wastewater published in previous reports is comparedwith TiO_2_/MnO_2_ PVC photocatalytic membrane in this work illustrated that TiO_2_/MnO_2_ PVC photocatalytic membrane possessed an excellent property, indicating clearly that this high-function modified membranes can expand the level of industrial wastewater treatment technique.

**Table 4:** Different methods used in pulp and paper wastewater published in previous reports is compared with TiO_2_/MnO_2_ PVC membrane.

Method	Parameters	Rejection (%)	Ref
Forward osmosis: Cellulose triacetate (CTA)	Lignin and COD	90 and 64	^111^
Catalysis : iron on natural zeolites (Fe/NZ)	COD and colour	71, and 88	^112^
Advanced Oxidation Process (AOP): Photo-Fenton and Peroxydisulfate PDS/UV	COD	75.5	^113^
Coagulation-flocculation: Alum and pomegranate seeds	COD and turbidity	81, and 98 A	^114^
Coupled advanced oxidation and biodegradation: ozone and TiO_2_	COD, BOD, and TOC	80.7, 91.6, and 84.6	^115^
Activated sludge bioreactor system: rice straw-activated carbon as biosorbent	COD	(63.2- 88.4)	^116^
Physicochemical and biological processes: Microbial communities integrated physicochemical	COD	99.8	^117^
Physicochemical treatment with an ozonation unit	COD	81	^118^
Dielectric barrier discharge ionization and a mixture of coagulants (poly aluminum chloride (PAC) and chitosan	COD	89.8	^119^
Coupled solar photo-Fenton followed by biological proces	COD and colour removal	98 and 95	^120^
Immobilizing white rot fungi to corn cob	COD and colour removal	50 and 80	^121^
Photocatalytic membrane: PVC blended with TiO_2_/MnO_2_	COD, TDS,Turbidity, and ABS	84 ± 0.4, 72 ± 0.2 91 ± 0.8,and 68.2 ± 1	Current study

## Conclusion

Using a natural plant extract, green TiO_2_ and MnO_2_ NPs were effectively created. The TiO_2_/MnO_2_ photocatalytic membrane was successfully created as a flat sheet using the phase inversion approach. Based on the hydrophilicity analysis, the research findings indicate that with an established contact angle of 68 TiO_2_/MnO_2_ photocatalytic membrane, the hydrophilicity of the membrane’s surface was increased by the nanocomposite PVC membrane. The membrane’s finger-like pore formation has been replaced by a macro-void and 83 percent porosity. The membrane’s remarkable capacity to remove humic acid from feed water through photodecomposition was demonstrated when exposed to solar light, increasing the flow rate. Membrane separation and photodegradation may be carried out simultaneously in the produced TiO_2_/MnO_2_ photocatalytic membrane thanks to the combination of membrane processing with the green heterogeneous photocatalyst. Removal of humic acid from wastewater was 98% successful. Paper and pulp effluent were purified in the dark and in the daylight, where the COD dropped from 1500 mg/L to 1015 mg/L (50%) and 247 mg/L (70%), respectively. Furthermore, in the dark and the sun, the TDS dropped from 1630 to 1200 and 452 ppt, respectively. This study clarifies how solar energy might enhance membrane separation and clean effluent from the pulp and paper industry. A streamlined system setup, financial gains, and environmental sustainability are just a few of the benefits that come with this integration. An alternative source for the sun was selected to facilitate the creation of a photocatalytic membrane that was cheap and did not require large areas**.**

## Data Availability

The datasets generated during and/or analyzed during the current study are available from the corresponding author upon reasonable request.
